# A case of retroperitoneal abscess caused by infection of urachal remnant

**DOI:** 10.1002/ccr3.5750

**Published:** 2022-04-20

**Authors:** Akira Yoneda, Taiji Hida, Hanako Tetsuo, Saeko Fukui, Shunsuke Murakami, Takayuki Miyoshi, Tatsuya Okamoto, Amane Kitasato, Hiroaki Takeshita, Tamotsu Kuroki

**Affiliations:** ^1^ Department of Surgery National Hospital Organization Nagasaki Medical Center Omura Japan

**Keywords:** retroperitoneal abscess, urachal remnant, ureteral resection

## Abstract

Infection of urachal remnant may cause recurrent abscesses. In the current case report, we describe a urachal remnant infection leading to a retroperitoneal abscess, which is an extremely rare condition. In such cases, the recommended treatment is urachal remnant resection.

## Abstract 2

The urachal remnant infection leading to a retroperitoneal abscess is an extremely rare condition. Urachal remnant resection is the recommended treatment for radical cure.

## 1 CASE PRESENTATION

The infection of urachal remnants is usually localized. A retroperitoneal abscess resulting from such infection is rare..^1^, ^2^

**FIGURE 1:**
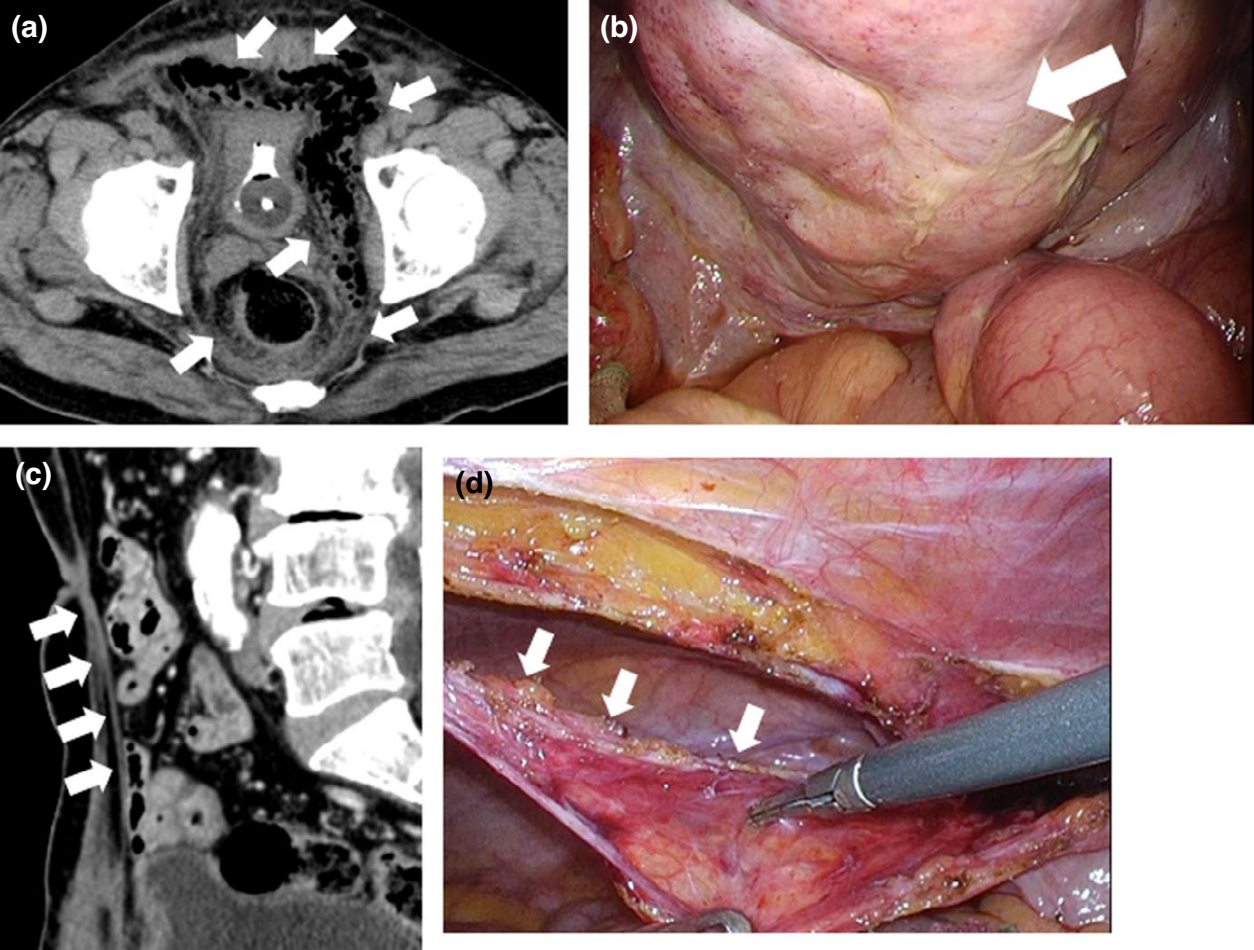
(A) CT. Gas and extensive abscess formation in the preperitoneal space from the lower abdomen to the pelvis. (B) Laparoscopic image. There was marked swelling of the abdominal wall centered on the midline umbilical cord, suggesting abscess formation in the same area. (C) CT. A slightly hyperdense cord‐like structure extending from the umbilical region to the bladder, which was assumed to be a urachal remnant. (D) Laparoscopic image. The median cord, i.e., the presumed urachal remnant, was detached from the abdominal wall

A 65‐year‐old man with fever and abdominal pain was admitted to our hospital. Complete blood count and chemo panel revealed a high inflammatory reaction with 11,200/ul white blood cells and 42.5 mg/L C‐reactive protein. Computed tomography (CT) revealed gas and abscess formation in the preperitoneal space (Figure 1A). We performed an emergency laparoscopic operation. Laparoscopic examination revealed swelling of the abdominal wall centered on the midline umbilical cord, suggesting abscess formation (Figure 1B). The Retzius cavity was opened, and a drainage tube was placed. The microbiological profile was anaerobic gram‐negative bacteria. At 6 months postoperatively, a recurrence of retroperitoneal abscess was observed. The patient underwent CT‐guided drainage of this abscess. CT showed a hyperdense cord‐like structure extending from the umbilical region to the bladder assumed to be a urachal remnant (Figure 1C). This led us to the conclusion that the abscess must have formed in this urachal remnant, subsequently spreading to the retroperitoneum. A laparoscopic ureteral remnant resection was performed. The median cord, that is, the presumed urachal remnant, was detached from the abdominal wall (Figure 1D).

## CONFLICT OF INTEREST

The authors declare that there is no conflict of interest that could be perceived as prejudicing the impartiality of the research reported herein.

## AUTHOR CONTRIBUTION

Akira Yoneda involved in case identification, writing, review, and editing the manuscript. Taiji Hida, Hanako Tetsuo, Saeko Fukui, Shunsuke Murakami, Takayuki Miyoshi, Tatsuya Okamoto, Amane Kitasato, and Hiroaki Takeshita involved in review, writing, and editing the manuscript. Tamotsu Kuroki involved in supervision of the study.

## ETHICAL APPROVAL

The study is conducted ethically in accordance with the World Medical Association Declaration of Helsinki.

## CONSENT

Informed consent for publication and related images has been obtained from the patient.

## Data Availability

The data that support the findings of this study are available on request from the corresponding author. The data are not publicly due to privacy or ethical restrictions.
